# The crystal structure of NS5A domain 1 from genotype 1a reveals new clues to the mechanism of action for dimeric HCV inhibitors

**DOI:** 10.1002/pro.2456

**Published:** 2014-03-18

**Authors:** Sebastian M Lambert, David R Langley, James A Garnett, Richard Angell, Katy Hedgethorne, Nicholas A Meanwell, Steve J Matthews

**Affiliations:** 1 Department of Biological Sciences, Centre for Structural Biology, Imperial College LondonSouth Kensington, London, SW7 2AZ, United Kingdom; 2 Bristol-Myers Squibb Research and Development, Computer Assisted Drug DesignWallingford, Connecticut, 06492; 3 UCL, Translational Research OfficeLondon, W1T 7JA, United Kingdom; 4 Bristol-Myers Squibb Research and Development, Discovery ChemistryWallingford, Connecticut, 06492

**Keywords:** hepititis virus, genotype 1a, NS5a, crystal structure, Daclatasvir

## Abstract

New direct acting antivirals (DAAs) such as daclatasvir (DCV; BMS-790052), which target NS5A function with picomolar potency, are showing promise in clinical trials. The exact nature of how these compounds have an inhibitory effect on HCV is unknown; however, major resistance mutations appear in the N-terminal region of NS5A that include the amphipathic helix and domain 1. The dimeric symmetry of these compounds suggests that they act on a dimer of NS5A, which is also consistent with the presence of dimers in crystals of NS5A domain 1 from genotype 1b. Genotype 1a HCV is less potently affected by these compounds and resistance mutations have a greater effect than in the 1b genotypes. We have obtained crystals of domain 1 of the important 1a NS5A homologue and intriguingly, our X-ray crystal structure reveals two new dimeric forms of this domain. Furthermore, the high solvent content (75%) makes it ideal for ligand-soaking. Daclatasvir (DCV) shows twofold symmetry suggesting NS5A dimers may be of physiological importance and serve as potential binding sites for DCV. These dimers also allow for new conformations of a NS5A expansive network which could explain its operation on the membranous web. Additionally, sulfates bound in the crystal structure may provide evidence for the previously proposed RNA binding groove, or explain regulation of NS5A domain 2 and 3 function and phosphorylation, by domain 1.

## Introduction

Hepatitis C Virus (HCV) is expected to be an increasing global healthcare concern.^1^ HCV is a major cause of chronic hepatitis, liver cirrhosis and hepatocellular carcinoma (HCC) and is estimated to infect up to 3% of people worldwide.^2^ No protective vaccine currently exists against HCV and the most common combination therapy based on pegylated-interferon α2 and ribavirin are effective in only a fraction of patients and these are plagued by adverse effects,^3^ even in combination with new drugs such as telaprevir (Vertex Pharmaceuticals) and boceprevir (Merck & Co.).^4^,^5^ The HCV species is highly genetically diverse and classified into six genotypes (1–6) with more than 90 different subtypes. Genotypes 1a and 1b are the most common, accounting for about 60% of global infections.^6^ The distribution of HCV genotypes varies globally and this heterogeneity affects virus pathogenicity and their responsiveness to therapies (WHO, 2003). Hence, current efforts being pursued in the treatment of HCV are focused on the development of direct acting antivirals (DAAs).^7^ The HCV genome encodes 10 proteins–consisting of the structural proteins; core, E1 glycoprotein and E2; and the nonstructural viral proteins p7, NS2, NS3, NS4A, NS4B, NS5A, and NS5B.^8^ All of the HCV proteins are essential for efficient HCV propagation and are therefore all potential targets for antiviral compounds. The recently approved NS3–4A protease inhibitors, telaprevir and boceprevir, used in combination with the established interferon and ribavirin therapy have created a new standard of care for patients with genotype 1 infections.^9^ In addition, DAAs showing high potency against the functionally well-characterized NS5B polymerase are already showing promise in phase III clinical trials,^10^ while HCV proteins p7, NS4B, and NS5A are currently under investigation as therapeutic targets.^11^ The continued development of combination therapies with more effective and tolerable DAAs that also allow the elimination of interferon-based regimes will be fundamental in the future successful treatment of HCV infection.^12^

NS5A is a 447 amino acid phosphoprotein of 56/58 kDa implicated in a variety of roles from the replication of HCV RNA, the modulation of the host cell responses and the assembly of viral particles.^13^–^15^ Despite this critical and expansive role in the virus life cycle, no specific mechanism is yet ascribed to NS5A. Domain 1 of NS5A consists of a zinc-binding motif^16^ and an N-terminal amphipathic helix which is necessary and sufficient for association of NS5A with the ER membrane^17^ and the structure is essential for HCV genome replication.^18^ Domains 2 and 3 appear to form an intrinsically unstructured region of the protein with roles in RNA replication and the assembly of hepatitis C virus particles.^15^,^19^,^20^

**Figure 1:**
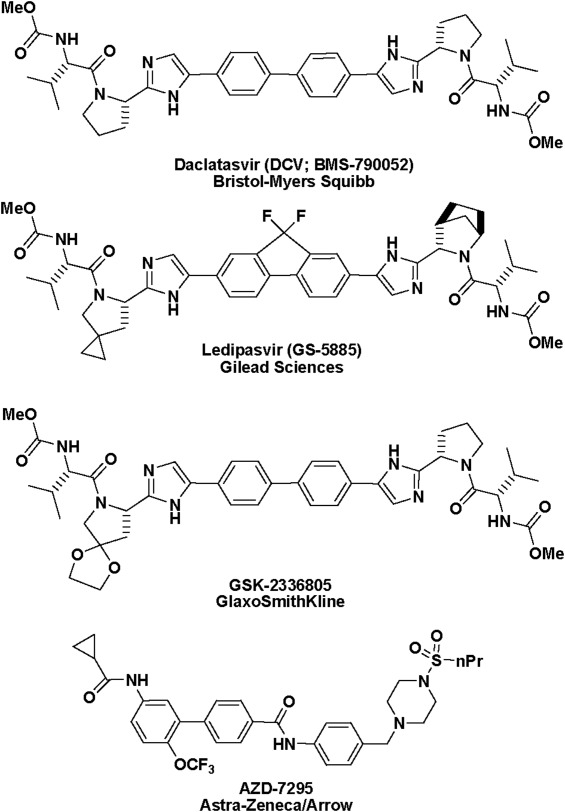
Chemical structure of the family of NS5A-targeting molecules.

The pivotal role of NS5A in virus replication makes it an ideal target for inhibition. Recent high-throughput screens (HTS) for inhibitors of HCV replicon propagation yielded leads that were optimized into compounds with picomolar inhibitory potency Fig. 1. To date, no exact binding data have been validated, although resistant mutant analysis has revealed several mutations within domain 1 of the NS5A gene that confer sensitivity to inhibitors^21^ and mono- and bis-crosslinking of a photoaffinity probe with the N-terminal region of NS5A^22^ has been demonstrated. An example of such a compound is daclatasvir (DCV, Bristol-Myers Squibb, Wallingford, CT), which is the representative of a mechanistic class that exhibits picomolar half maximal effective concentrations (EC_50_) towards HCV replicon cell cultures^23^ with the twofold symmetry playing an important role in its effectiveness, particularly with respect to pan-genotype inhibition. More specifically, 1a genotypes show lower sensitivity (approximately 5- to 10-fold lower) than the 1b genotypes with symmetrical or pseudo-symmetrical compounds and resistance mutations generated in genotype 1a replicons result in a greater increase in resistance (shifts in EC_50_ values approximately 2 magnitudes greater) than those conferred from similar mutations in genotype 1b.^24^ However, smaller, monomeric compounds can exhibit potent activity towards genotype 1b replicons but a much reduced potency towards genotype 1a, initially viewed as a hallmark of the NS5A inhibitor class.^25^

Crystal structures of genotype 1b NS5A domain 1 (NS5A-D1) have revealed the formation of dimers during crystallization. Although the formation of multimers or protein-protein contacts are inevitable consequences of protein crystallization,^26^ there is some *in vitro* evidence of dimerization^27^ and the phenomenal potency of this class of dimeric compounds has led to speculation that they act on a physiologically-relevant dimer present in NS5A-D1 crystal structures. While unproductive binding can occur,^22^ productive binding, hypothetically, over-stabilizes NS5A dimerization^21^ potentially in the form of an immature HCV polyprotein dimer, or higher order multimer, to slow the cleavage of the NS4B-NS5A precursor.^28^ This hypothesis is supported by the bis-crosslinking of NS5A by BMS-351, an inhibitor of HCV replication that incorporates photo-activated functionality^22^ and the dramatic improvement in potency obtained with symmetrical and pseudo-symmetrical inhibitors relative to half molecules, particularly with genotype 1a.^29^,^30^ The first crystal structure of a NS5A-D1 construct consisting of residues 25 to 215 from genotype 1b subtype Con1, revealed it to consist of two subdomains and to contain a novel fold with a stabilising disulphide bond. Additionally, the asymmetric unit comprised a dimer with an intermolecular interface of approximately 830 Å^2^, which forms a large groove that could accommodate viral RNA;^31^ this dimer has served as the basis for the working model of the NS5A-D1. Crystallization of a NS5A-D1 construct from the same genotype and subtype containing residues 33 to 202 resulted in higher resolution structures and showed that the NS5A-D1 formed an alternative dimer with a 910 Å^2^ dimeric interface. Through combining the two dimers, it was hypothesized how a NS5A superhelical array could form.^32^ The first crystal form does not seem to be readily reproducible and soaks of the second crystal form with compounds has yet to yield structures of ligand-bound complexes (unpublished data).

Neither crystallographic nor spectroscopic evidence for compounds such as DCV binding to NS5A-D1 is currently available although binding of a structurally related affinity probe to genotype 1b NS5A has been demonstrated.^23^ To address this and investigate the differences between the compound's effect on genotypes 1b and 1a, we decided to determine the structure of NS5A-D1 from genotype 1a. Our crystals of domain 1 from genotype 1a NS5A show a new crystal form, and allowed structural determination via X-ray crystallography to a resolution of 3.5 Å. Intriguingly, our structure shows two new dimeric forms of this domain. NS5A-D1 dimers allow the formation of new hypothetical multimer assemblies, which form an expansive network that could operate in the membranous web. Experimental observation of RNA binding^13^,^27^,^33^ support a hypothesis that sulfates from the crystallization buffer bound in the crystal structure may mimic a RNA backbone and highlight the location of a RNA binding groove. Alternatively, this region of NS5A may bind phosphorylated sites of NS5A domain 2 and 3 to alter its overall conformation and regulate progression from RNA synthesis to viral assembly: this may explain how DCV expresses two MOAs,^41^ affecting both viral replication and assembly, and blocking hyperphosphorylation of NS5A.

## Results

### Crystal structure of genotype 1a NS5A-D1

**Figure 2:**
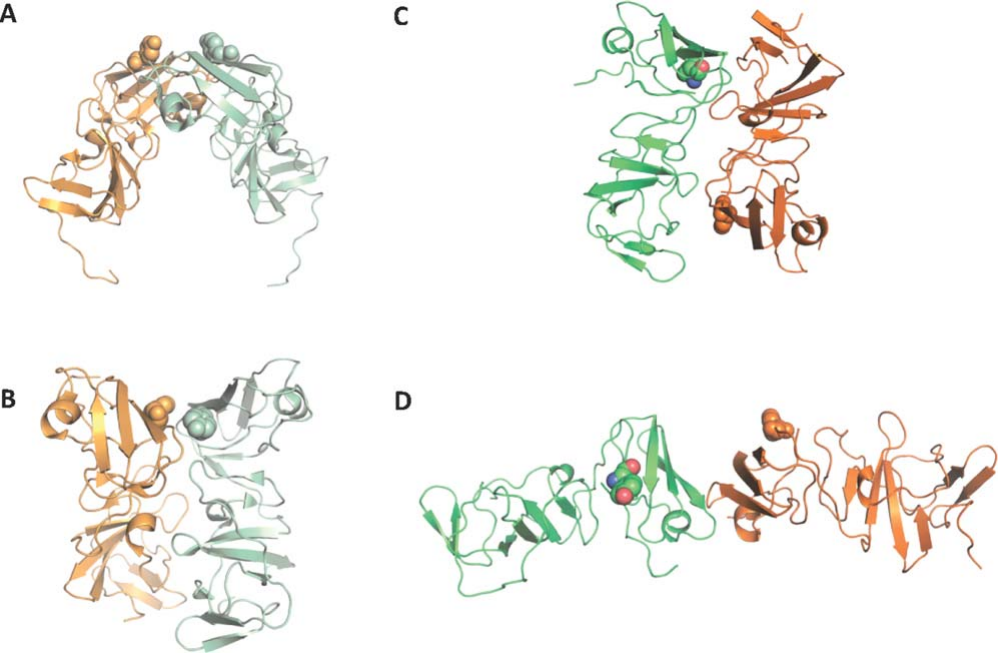
Genotype 1b and 1a NS5A domain 1 crystal structure dimers in ribbon representation. The side chain of tyrosine 93, a major resistant mutation, is displayed as spheres. Monomers are shown in orange and green, and coloured in pastel for genotype 1b dimers. (A) The first crystal structure dimer^31^ forms a potential RNA-binding pocket. (B) The second genotype 1b dimer^32^ are juxtaposed in parallel to form an extensive interface. (C) Monomers A and B from the genotype 1a NS5A domain 1 crystal structure share the same interface as the Love *et al*. dimer shown in B albeit with the monomers arranged in an antiparallel fashion. Use this link to access the interactive version of this figure. (D) Monomers C and D form a N-terminal, head to head dimer. Use this link to access the interactive version of this figure.

Our crystals of genotype 1a NS5A-D1 represent a new crystal form. The crystal structure consists of four monomers of the genotype 1a NS5A-D1 arranged into T-shaped asymmetric units (Fig. 2 and Supporting Information Fig. S1), which are juxtaposed to form large solvent channels; the crystals have a Matthews coefficient of 4.73 Å^3^/Dalton and a solvent content of 73.97%.^34^ Similar surfaces of the molecule are exposed to the solvent as in the most recent 1b NS5A Domain 1 dimer crystal structure.^32^

The overall fold of each monomer in our model superposes well with the NS5A domain structures from genotype 1b^31^,^32^ with an average Cα RMSD of 0.57 Å between 1a and 1b monomers. The zinc-binding motif has the same coordination as the genotype 1b NS5A Domain 1 with an average distance of 2.24 Å between coordinating sulphurs and the zinc atom. These bond lengths are slightly less than the ideal distance (2.35 ± 0.09 Å) for structural metal zinc-coordination sites and slightly more than the ideal distance (2.08 ± 0.13 Å) for catalytic metal zinc-coordination sites.^35^ The arrangement and proximity of residues Cys 142 and Cys 190 in our model and the presence of electron density in between the residues suggests the formation of a disulphide bridge, despite the presence of DTT in the crystallization buffer, with a distance between the sulfur atoms of 2.0 Å, 2.0 Å, 2.0 Å, and 2.5 Å in monomers A, B, C, and D, respectively. It is not known whether this disulphide bond is physiologically important or essential for HCV replication.^16^

Amino acids present in the construct that could not be fitted to any electron density include the disordered N- and C-termini of three to four amino acids and 13 to 16 amino acids, respectively, depending on the monomer. Loops between β-strands B7 and B8, and β-strands B8 and B9 could not be fully modeled in monomer A, in which modeling of residue Glu 172, and residues Gly 178 and Leu 179, respectively, had to be omitted.

Situated in between monomers A, C and D, there was a large unmodeled region in the electron density difference map appearing to be coordinated by nitrogens ε from residues Arg41 and Arg81 from monomer A and by a nitrogen η from Arg78 from monomer B. Owing to the presence of these positively-charged side-chains and the high concentration of sulfate ions in the crystallization solution, it was modeled as SO_4_^2−^ (Supporting Information Fig. S2A). Arg41 and Arg 78 are strictly conserved as positive amino acids (>90% Arg or Lys at position 41 and >95% Arg or Lys at position 78), whereas Arg81 shows no conservation (Arg, Ser, Ala, Met, or Leu substitutions at position 81). Although the electron density matched that expected for a sulfate ion, we cannot rule out that this site is occupied by another anion. Another region of electron density was identified adjacent to monomer A but was not modeled as it was less well defined. Nevertheless, it shows potential coordination by Arg 44 from chain C and the backbones of Gly 45 and Met 72 from chain A and we tentatively propose that this region includes an additional sulfate ion (Supporting Information Fig. S2B).

### Dimer interfaces

**Figure 3:**
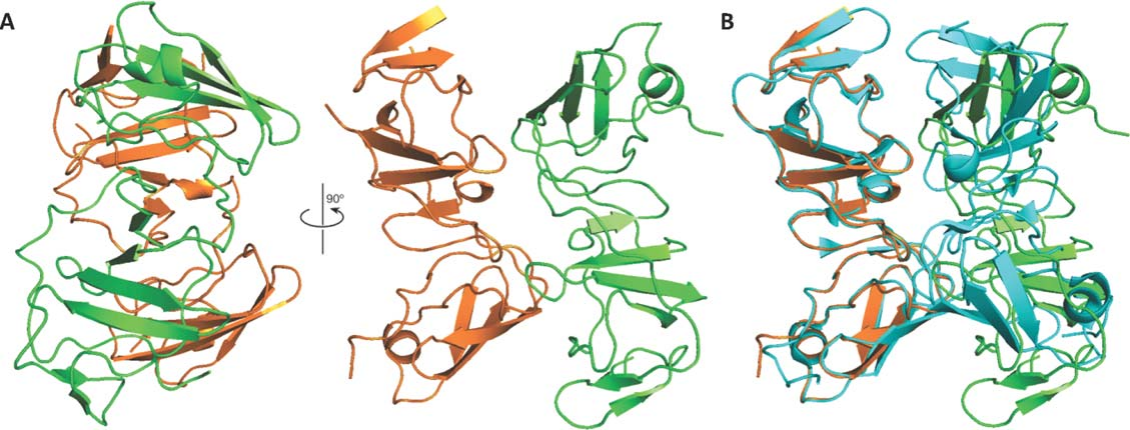
(A) Monomers A (orange) and B (green) make up dimer AB. (B) A secondary structure alignment of monomer A from our genotype 1a crystal structure with monomer B from the Love *et al*. dimer (blue). Dimer AB shares a similar dimer contact face to the Love *et al*. dimer except it is less closely packed and in a head to tail conformation.

Two new dimeric forms of NS5A-D1 are observed from the crystal packing shown in the asymmetric unit. Monomers A and B of our structure are related by a twofold axis (179.9°) perpendicular to their length. Dimer AB displays a similar interface to that previously reported,^32^ but one of the monomer's orientation is reversed creating a head-to-tail conformation [Fig. 3(A)], rather than head-to-head. Dimer AB has a smaller buried solvent-accessible surface area of 435.1 Å^2^ than that calculated for the Love *et al*.^32^ dimer (957.1 Å^2^), due to the more detached packing of the two molecules—the distance between the centers of mass of the monomers in dimer AB is 23.1 Å compared to 21.4 Å in the Love *et al*. dimer [Fig. 3(B)]. The contact surfaces are formed by residues 97–99, 112–115, 149, 160–161. Amino acid residues Pro 97, Cys 98, and Thr 99 show low conservation and are in contact with the conserved residue Phe 120 of the other dimer. Conserved amino acid residues Arg 122, Val 123, Ala 125, and the non-conserved Ser 124 form contacts with conserved residues Arg 160 and Phe 161 (which is Tyr or His in other genotypes). All of these 10 residues are also included in the contact area of the Love *et al*. dimer, whose contact area also includes an additional 14 residues. Pairs of residues forming notable interactions reported in the Love *et al*. dimer, such as the salt bridge formed by conserved residues Glu 148-Arg 112, the backbone hydrogen-bond between conserved residues Ala 92 and Gly 96, and an electrostatic interaction between residues Arg 48 and Glu 116, are not close enough to form in dimer AB.

**Figure 4:**
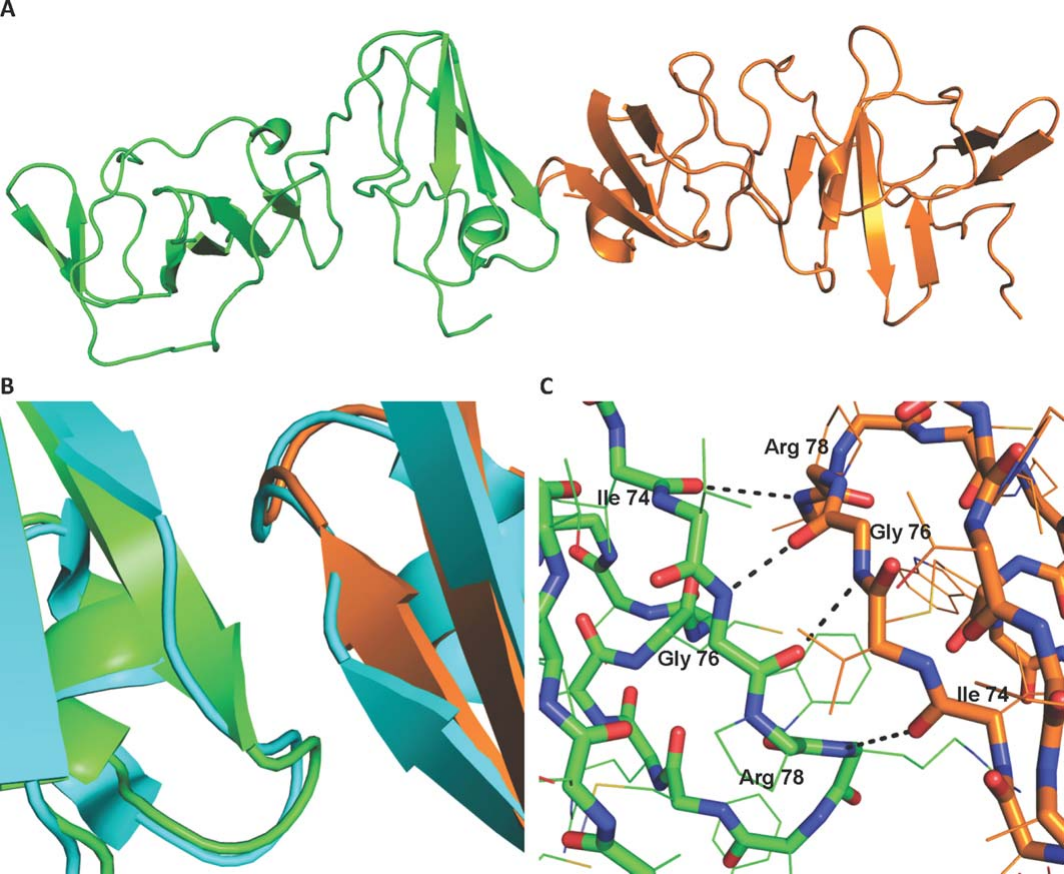
(A) Monomers C (orange) and D (green) make up dimer CD. (B) A secondary structure alignment of monomer A from the Love *et al*. dimer (blue) with each monomer C and D shows an extension of β-strand B3 from Val 75 to Arg 78. (C) Hydrogen bonds are formed between residues Ile 74 O and Arg 78 NH, Gly 76 NH and Gly 76 O, forming a total of four hydrogen bonds in the CD dimer.

Monomers C and D form a second dimer CD that shows an approximately twofold (173.6°) symmetry axis, composed of the monomers aligned in a head to head conformation [Fig. 4(A)]. The monomers form a buried solvent-accessible surface area of 315.0Å^2^. The contact surfaces are formed by residues 74 to 78 and 83 to 84. Residues 74 to 78 from each molecule appear to form an intermolecular anti-parallel β-sheet through an extension of β-strand B3 from Val 75 to Arg 78 [Fig. 4(B)]. The formation of this intermolecular β-sheet does not disrupt the β-sheets within each molecule. All the residues involved in the intermolecular β-sheet Ile 74 to Arg 78 are conserved. Residues 83 to 84 from each molecule may stabilize the dimer through additional van der Waals interactions. Met 83 shows conservation as a hydrophobic residue (>99% residues are Met, Thr, Val, or Ile) while Trp 84 is highly conserved (>95% Trp). In forming the intermolecular β-sheet, the refined structure shows intermolecular backbone hydrogen bonds between pairs of residues Ile 74 O with Arg 78 NH, and Gly 76 NH with Gly 76 O. Copies of dimer CD are packed together in the crystal with the same interface observed in dimer AB.

### Simulation of the NS5a CD-dimer/DCV complex

**Figure 5:**
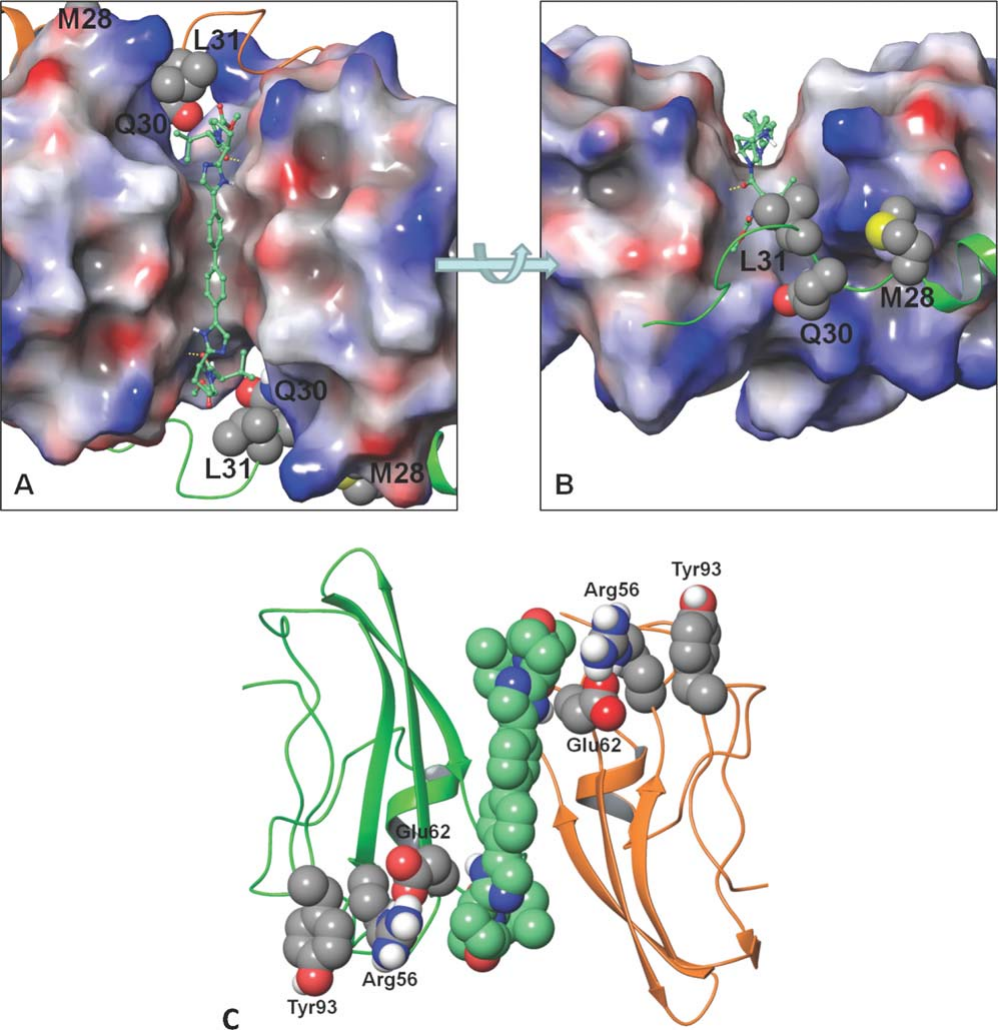
DCV docked into the cleft between the CD dimer. The surface of the dimer is coloured by its electrostatic potential (red is negative, gray is neutral, and blue is positive). The N-terminal residues are shown in ribbon (C-domain in green, D-domain is orange). Key resistant positions are shown with van der Waals surface (carbon: gray, nitrogen: blue, oxygen: red, sulfur: yellow, polar hydrogen: white). DCV is shown in a ball and stick depiction (carbon: aquamarine). A) Top view of DCV docked symmetrically through the cleft. B) Side view focuses on the cap of DCV docked into the cleft. Yellow dotted lines indicate the hydrogen bond between the DCV valine carbonyl and the NS5A Glu62 backbone amide NH. Note: L31 packs against the cap valine moiety of DCV. C) Communication network between DCV and Tyr93. The ribbon depiction of the CD monomer is shown in green and orange, respectively. The van der Waals surface of the DCV (carbon: aquamarine) and the Glu62, Arg56, and Tyr93 (carbon: gray) communication network. Use this link to access the interactive version of this figure.

The physical chemical properties and dimensions of the cleft between the head-to-head CD-dimer match well with that of the DCV molecule. Although crystal soaking experiments with DCV failed, this is not surprising as the amphipathic helix and its linker is absent in our structure. The high frequency of escape mutants occurring in this region suggests an active role drug binding. We therefore performed molecular dynamic simulations to add the experimental NMR structure of the N-terminal helix to our structure and model DCV into the interdimer cleft. In the final modeled structure the central floor region of the cleft is composed of hydrophobic residues Val75, Gly76, and Pro77 which lay under the biphenyl core and between the caps of DCV while the periphery of the putative binding site contains more polar residues including His54, Arg56, Glu62, His66, Arg73, and Thr79 which are proximal to the cap regions of DCV (Fig. 5). The only hydrogen-bonds between DCV and the NS5A CD-dimer observed in the docked model are between the backbone NH's of Glu62 and the two DCV valine carbonyls (Fig. 5). To further evaluate the putative binding model, a 40 ns MD simulation was conducted. The model drifted slightly from the starting structure over the heating and equilibration phase and then stabilized (Fig. 5). Each monomer and DCV remained stable over the production phase; however, the RMSD for the CD-dimer shows a slow oscillation as the structure fluctuates about the long symmetry axis (see movie, supplemental material). The mean distance between DCV and the amino acids that line the putative binding cleft within the CD-dimer are shown in Supporting Information Table I. The two hydrogen-bonds observed between DCV and the dimer did not remain constant but periodically formed and broke throughout the simulation.

**Table I:** Crystal Data Collection and Refinement Statistics

Crystal parameters	
Space group	P2_1_2_1_2_1_
Cell dimensions	*a* = 100.25 Å; *b* = 101.53 Å; *c* = 148.70 Å
Molecules per asymmetric unit	4
Data collection	
Beamline	DLS I24
Wavelength (Å)	0.97780
Resolution range (Å)	51.475–3.5 (3.67–3.5)
Unique observations^a^	17168 (1226)
R_merge_^a^	0.135 (0.388)
<*I*>/σ*I*^a^	3.5 (1.9)
Completeness (%)^a^	86.0 (87.5)
Multiplicity^a^	3.4 (3.3)
Refinement	
*R*_work_/*R*_free_(%)	22.49/26.64
No. protein residues in monomer	159
RMSD stereochemistry	
Bond lengths (Å)	0.0147 (monomer A)
Bond angles (°)	2.453 (monomer A)
RMSD of monomers	
B to A (Å)	0.412
C to A (Å)	0.454
D to A (Å)	0.622
PDB accession code	4CL1

a PDB, Protein Data Bank. Values reported in the format: overall data (last resolution shell). RMSD (root-mean-square deviation) stereochemistry is the deviation from ideal values.

**Table II:** Summary of NS5A Domain 1 Crystal Structure Dimer Properties

Dimer	Distance between the centers of mass of the monomers (Å)	Buried (solvent-accessible surface) area (Å^2^)	Rotation angle (°)
Tellinghuisen 1b	25.0	1022.1	178.1
Love 1b	21.5	957.1	180.0
1a AB	23.1	435.1	179.8
1a CD	48.5	315.0	173.7

The most frequently observed, clinically-relevant NS5A genotype 1a (GT-1a) resistance mutations have been identified at amino acids 28, 30, 31, and 93 and the linked positions Q30R-E62D, whereas for GT-1b, the major resistant changes occurred at residues 31 and 93.^36^ The key resistance mutation that is present in our structure is Tyr93. Although this residue does not reside directly within the putative binding site, it is connected to DCV via a communication network in the MD simulation. During the course of the simulation, DCV intermittently hydrogen-bonds with Glu62 via the backbone amide NH to the DCV valine carbonyl or the Glu62 side-chain and the DCV imidazole NH; Glu62, in turn, periodically forms a salt bridge with Arg56 which packs against Tyr93 to complete the network. This allosteric mechanism of resistance is supported by the recent demonstration that resistant NS5A does not necessarily eject an inhibitor and that binding of an inhibitor to NS5A alone is not sufficient for inhibition.^22^ It is worth noting that mutations are often to smaller or more flexible residues, which may allow NS5A to recover its ability to articulate in the presence of an inhibitor leading to a more flexible dimer interface or allowing NS5A to populate other dimer structures that are required for function. Interestingly, the communication network between Tyr93 and DCV also contains Glu62, a position that has been associated with resistance. However, Glu62 resistance normally arises when linked with the primary Q30R mutation.^36^

## Discussion

It has been established that dimeric inhibitors, either symmetrical or pseudo-symmetrical, are a key factor in the antiviral activity in their compounds such as DCV, which shows 50 and 9 pM EC_50_ values against 1a and 1b HCV replicons, respectively. Key resistance mutations are generated at Leu 31 and Tyr93 in both genotypes and additionally at Met28 and Gln30 in genotype 1a.^23^ Similar resistance profiles are generated in response to treatment with structurally related compounds including BMS-766 (Bristol-Myers Squibb), Ledipasvir/GS-5885 (Gilead Sciences), GSK2336805 (GlaxoSmithKline), PPI-461 (Presidio, structure not disclosed) as well as AZD7295 (AstraZeneca/Arrow Therapeutics) which represents a distinct chemotype that only weakly inhibits genotype 1a HCV (Fig. 1). The crystal structure the genotype 1a NS5A reveals that these crystals have very high solvent content. Our new crystal form is therefore likely to be amenable to soaks with large compounds and would serve as an additional tool in the development of novel NS5A-targeted therapeutics.

The current working hypothesis is that such compounds act on an NS5A dimer, based on the dimeric nature of DCV analogs, the location of NS5A resistance mutations, and mono- and bis-crosslinking data, albeit with a lack of crystallographic or spectroscopic evidence. Publication of the crystal structure for two genotype 1b dimers^31^,^32^ has caused debate as to which dimer is biologically relevant or whether several dimer forms are of importance. Our new results provide further candidates for physiologically-relevant NS5A dimers. Due to the dimensions of the compounds targeting NS5A, we deem that the head to tail conformation of dimer AB places the two N-termini too far apart, although this does not rule out another physiological role for dimer AB. The monomers of dimer CD have adjacent N-termini and this dimer could represent a potential target of the compound. Although dimer CD has a relatively small buried solvent accessible surface area of 315.0 Å^2^, the inclusion of the N-terminal amphipathic helix *in vivo* may further stabilize its formation. It is conceivable that DCV may disrupt the HCV life cycle by stabilizing the formation of a weak dimer such as the dimer CD.

Through the use of molecular docking and molecular dynamics we have shown that the binding cleft between the head-to-head CD-dimer could accommodate DCV in a fashion such that the key resistance mutations lie in proximal to the inhibitor. This demonstrates the CD-dimer could serve as a potential binding site for DCV in addition to the previous models which show DCV could interact with genotype 1b dimers.^22^ Specifically, the two mutations observed in our structure (Y93H and E62D) are within two amino acid layers of DCV and establish a communication network through R56. However, based on the relative strength of the Y93H and E62D resistance that is observed, we cannot rule out that other binding modes are possible as DCV has been shown to complement the symmetry of the previous NS5A dimers close to primary clinical resistance sites. Binding poses of BMS-411with the face-to-face NS5A domain I dimer structure from Tellinghuisen *et al*.^31^ and GSK2236805 with the back-to-back Love *et al*. structure^32^ have been previously proposed.^22^,^37^,^38^ As with our dimer model reported herein, the amino-terminal amphipathic α-helix was modeled using the NMR structure by Penin *et al*.^39^ All the models suggest a symmetric binding mode along the dimer interface where inhibitors can interact with regions proximal to resistance mutations at positions at 28, 30, 31, 54, and 93 and genotype 1a/1b changes at positions 28, 30, 54, 56, 58, and 62. While the Love *et al*. dimer provides a cleft for binding with good shape complimentarily to the NS5A inhibitors, the chemical nature of the cleft is reversed from that of the inhibitors. Specifically, the hydrophobic biphenyl core of the inhibitor is positioned over the two adjacent T95 amino acid residues at the center of the dimer interface and the polar imidazole moieties sit over the hydrophobic aromatic ring of the Y93 side chains. A better match is seen in the BMS model where the inhibitor core lies over the two adjacent F37 residues at the dimer interface and the imidazoles are positioned for hydrogen bonding with Q62 from each monomer and the N-terminus is placed adjacent to the inhibitor. However, it should be noted that, unlike our dimer, this crystal structure does not present a cleft for inhibitor binding in the absence of the modeled in N-terminal helix. While each model has its merits, it is also conceivable that packing forces contribute to the formation of dimeric species during crystallization and these may not be physiologically significant. Our preferred explanation is that NS5A binds a variety of cellular targets and these dimers explain some of the multiple interactions of NS5A. Additional mutational and structural studies are needed to elucidate the precise details of the NS5A/inhibitor complexes.

The discovery of our new NS5A dimers suggests that the formation of an extended multimeric network of NS5A may occur through the utilization of various dimer interfaces. The combination of the two genotype 1b NS5A-D1 dimers allowed the formation of a superhelical array that provides a model for the oligomerization of NS5A and NS5A-D1 constructs observed *in vitro*. However, the formation of this higher order oligomer into a non-planar, superhelical array precludes its association with a lipid bilayer.^32^ By combining the different conformations of the new NS5A-D1 dimers present in our crystal structure, one can envisage an array of NS5A molecules that forms a more expansive network that could interact with the membranous web (Supporting Information Fig. S3). NMR experiments of NS5A constructs have shown the protein can reversibly aggregate in a concentration dependent manner (data not shown), possibly into ordered oligomeric states. Such complexes could imaginably have roles in the formation of the membranous web, the replication complex, and/or the assembly complex on lipid droplets.

**Figure 6:**
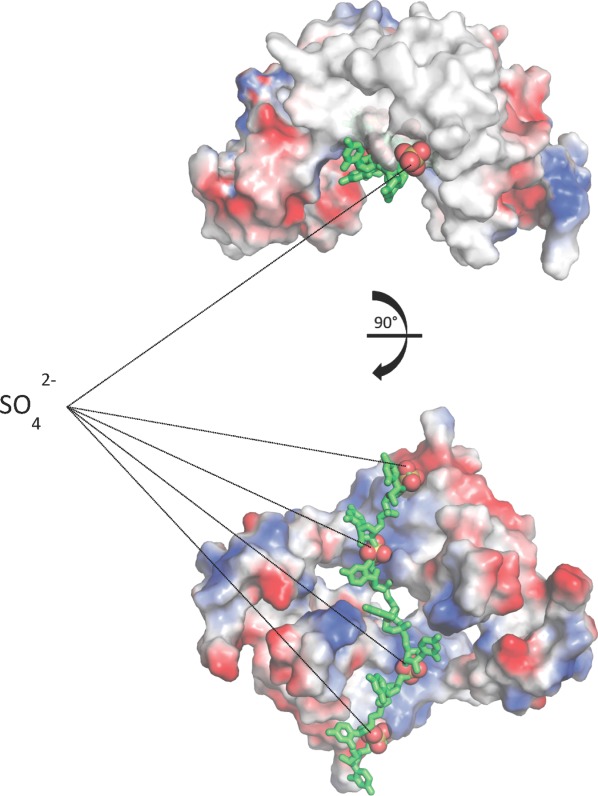
Viral RNA approximately modeled into NS5A domain 1 dimer. A structural alignment of monomer A and its associated sulfates from our genotype 1a crystal structure with each monomer in the Tellinghuisen *et al*. dimer places the sulfates in the dimer's RNA-binding groove. The sulfates may be mimicking an RNA phosphate backbone allowing the modeling of viral RNA into the dimer. Use this link to access the interactive version of this figure.

As SO_4_^2−^ and the phosphate from an RNA backbone have similar conformations and charge, it could potentially mimic the natural RNA ligand of NS5A. Interestingly, while the sulfate bound within our crystal structure performs a stabilizing role in crystal formation, a structural alignment of monomer A, which coordinates the SO_4_^2−^ via residues Arg 41 and Arg 81, with the Tellinghuisen *et al*. genotype 1b structure places the anion in their proposed RNA-binding groove^31^ (Fig. 6). Alignments with other NS5A domain 1 dimers do not place the SO_4_^2−^ in any recognizable RNA binding groove. Assuming these to represent physiological RNA binding sites, it is possible to accommodate the approximate position of viral RNA (7rU) in the proposed RNA-binding groove of the Tellinghuisen *et al*. structure^31^ suggesting this “arched” dimer may be relevant for RNA binding by NS5A in the HCV life cycle (Fig. 6).

Another possible role for this binding site could be to sequester the phosphorylated C-terminus of NS5A. Phosphorylation of NS5A is thought to regulate its interaction with a variety of host and viral binding partners, hence regulating HCV replication and assembly. Therefore binding of a phosphorylated residue within the C-terminal would alter NS5A specificity for target host proteins and progression through the viral life cycle. This could explain how the targeting of NS5A inhibitors to NS5A-D1 could affect phosphorylation in domains 2 and 3, and halt the transition from viral RNA replication to virus assembly.^28^ Recently, the hyperphosphorylation of NS5A in domains 2 and 3 has been shown to be dependent on the phosphorylation of at least one residues in NS5A domain.^40^ This regulation of NS5A domains 2 and 3 by domain 1 could explain findings that have determined that DCV expresses dual MOAs: interfering with the roles of NS5A in both viral RNA synthesis and virion assembly,^41^ with the latter being an offshoot of NS5A-D1 dimer destabilization/stabilization.

Our current understand the mode of action for NS5A inhibitors centers on the disruption of the formation and function of the replication complex.^22^ It is thought that the compound-interaction site on the surface of NS5A is exposed only transiently during the HCV life cycle, which explains why interactions with NS5A have only been detected by treatment of whole replicons with compounds and not *in vitro*.^22^,^23^ The binding of inhibitors to NS5A perhaps during replication complex formation, could result in conformational changes to NS5A and the subsequent formation of non-functional replication complexes.^22^ The isolation of a NS5A dimer via chemical cross-linking of photoaffinity DCV analogs, provided the first direct evidence of compounds interacting with the N-terminal region of NS5A^22^ and emphasized the importance of dimerization of NS5A in the HCV life cycle and its inhibition. The structural distortion of NS5A by the DCV analogs may result in inhibition of the replication complexes through concerted communication through NS5A multimers.^42^ The NS5A domain 1 allows a variety of multimerized NS5A complexes to be built into an NS5A network. We suggest that all of the observed NS5A dimers could be physiologically relevant and involved in the formation of this network. Within this proposed network, at least one dimer mode would be the target of these dimeric DCV-like inhibitors leading to network disruption and malfunction of the replication complexes. As NS5A has multiple roles and NS5A inhibitors affect viral RNA synthesis and virion assembly,^41^ we suggest this proposed NS5A network also serves a purpose in viral assembly. The new dimer modes observed in the genotype 1a NS5A structure not only extends our knowledge of interactional surfaces that could play a role in the action of symmetrical NS5A inhibitors, but supports the notion that NS5A dimers play a critical physiological role in the viral life cycle.

## Materials and Methods

### Cloning and purification of 1a NS5A D1

Recombinant 1a (isolate H77) NS5A (residues 33–202) with a N-terminal TEV protease cleavage site (ENLYFQGSM) was expressed using the pET32 plasmid in BL21(DE3) *Escherichia coli* (Novagen) at 15°C for 16 h. Cells were harvested and resuspended in a wash buffer consisting of 100 m*M* Tris-HCl (pH 8.0), 300 m*M* NaCl, 10 m*M* imidazole, 10% glycerol, in addition with the protease inhibitors aprotinin at 2 μg/mL, leupeptin at 10 μ*M*, pepstatin at 1 μ*M* and phenylmethanesulfonylfluoride at 1 m*M* (Melford). The cell lysate supernatant was loaded onto a 5 mL HisTrap FF Crude (GE Healthcare) affinity column and washed with 100 ml of the wash buffer and eluted using the same buffer with 500 m*M* imidazole. The concentration of fractions containing the fusion protein was determined using a NanoDrop 1000 (Thermo Scientific) and the fractions were pooled and desalted into the wash buffer. One milligram of TEV protease (produced in-house^43^) per 100 mg of fusion protein was added for overnight cleavage at 20°C. After TEV protease cleavage, the His-thioredoxin-tag and TEV protease were removed from the cleaved sample by passing it through a second 5 mL HisTrap FF Crude (GE Healthcare) and collecting the flow-through and wash fractions, which were pooled and concentrated before injection onto a S75 column (GE Healthcare) equilibrated with a crystallization buffer consisting of 20 m*M* Tris HCl, 150 m*M* NaCl, 10% glycerol, pH 8.0, 5 m*M* dithiothreitol (DTT).

### Crystallization of 1a NS5A D1

Protein was concentrated to 8 mg/mL as determined with a NanoDrop 1000 (Thermo Scientific). To remove any precipitation, samples were subject to centrifugation at 16,060 *g* for 2 min. Initial hits were obtained from sparse matrix screens using sitting drop plates with 100 nL reservoir added to 100 nL protein with a mosquito crystallization robot (TTP LabTech) before sealing and incubation of the plates at 4°C. The largest crystals were grown using microseeding with the crystals from the original condition containing 0.1 *M* MES pH 6.5, 1.6 *M* MgSO_4_ which were crushed and used as a seeding stock for an optimization plate consisting of sitting drops of 2 µL of the protein sample with 2 µL reservoir. After 30 days incubation at 4°C, crystals were visible and grew to a full size of up to approximately 150 µm after 60 days.

### X-ray data collection and structure determination

Crystal hits were fished out of the solution in 0.1 to 0.2 mm cryoloops (Hampton), before being immersed briefly in a buffer consisting of 0.1*M* MES pH 6.5, 1.6 *M* MgSO_4_ and 30% glycerol, and then flash frozen in liquid nitrogen. Native data sets were collected using a PILATUS 6M detector at the micro focus beamline I24 at Diamond Light Source. Diffraction data was processed with xia2 (Winter, 2009) and phases were calculated with Phaser^44^ using the genotype 1b NS5A domain 1 structure (PDB code: 1ZH1) as a search model. The process of refinement using Phenix.refine with NCS restraints^45^ and model building was iterated. The model was validated using Molprobity and deposited within the PDB as 4CL1.^46^ Data collection and final refinement statistics are given in Table I. Buried solvent accessible surface area, rotation angles and distances between centers of masses of the dimers were calculated with Chimera and are summarized in Table II.^47^ Amino acid conservation at dimer contact sites was determined by 95% conservation from 25 sequences from genotypes 1 to 6 from the Los Alamos database.^48^ Figures were made using PyMOL (The PyMOL Molecular Graphics System, Version 1.5.0.4 Schrödinger, LLC.).

### DCV docking and molecular dynamics (MD)

The head-to-head CD-dimer from our structure was prepared for docking and molecular dynamics (MD) studies using the Schrödinger Protein Prep Wizard^49^ script. This script adds missing amino acid side chains and optimally adds hydrogen atoms into the structure. The cleft between the CD-dimer was identified as a potential binding site and Glide^49^ from the Schrödinger suite of programs was used to initially dock DCV into this pocket. The resulting docked structure of DCV was hand-adjusted to enhance the symmetry of the CD-dimer/DCV complex. The NMR structure of the N-terminal alpha helices (residues 1–31,^39^ 1R7C.pdb) was grafted onto each monomer of the CD-dimer/DCV complex. The missing residues (32–35) were modeled into place and restraints added to enhance symmetry of the final modeled structure.

### Molecular simulation details

The CD-dimer/DCV complex was placed in a 57.13 × 129.52 × 50.40 Å^3^ box with 9917 TIP3P^50^ water molecules and eight chloride ions. The MD simulation was carried out with NAMD.^51^ The Amber99SB^52^ force field was used to model the protein, solvent, and ions, while the General Amber^53^ force field was used for DCV. The Verlet integration scheme was used with a multi-time step^54^ of 2/2/6 and Shake^55^ was applied to all hydrogen bonds. The non-bonded interactions were tapered between 8 and 10 Å and particle-mesh-Ewald (PME)^56^ was used for long range electrostatics. The simulation was run with periodic boundary conditions in a NPT ensemble at 300 K and 1 atm. The solvated complex was first subjected to 250 steps of protein backbone-constrained conjugate gradient minimized followed by an additional 250 steps of unconstrained conjugate gradient minimization. This was followed by MD heating (10 K increments every 100 steps up to 300 K, NVT ensemble,) and equilibration (250 ps, NPT ensemble). The MD equilibrated system was subject to 40 nanoseconds (nsec) of MD and 40,000 frames, one frame per picoseconds (psec), were collected for data analysis.
