# Supplementary material for: The crystal structure of NS5A domain 1 from genotype 1a reveals new clues to the mechanism of action for dimeric HCV inhibitors
Source: Protein Sci. 2014 Mar 18;23(6):723–34. doi: 10.1002/pro.2456 (PMC4093949; doi:10.1002/pro.2456)
Supplement: Supplementary file 1 — Supplementary Information [file pro0023-0723-SD1.docx]

**Supplementary Information**

| 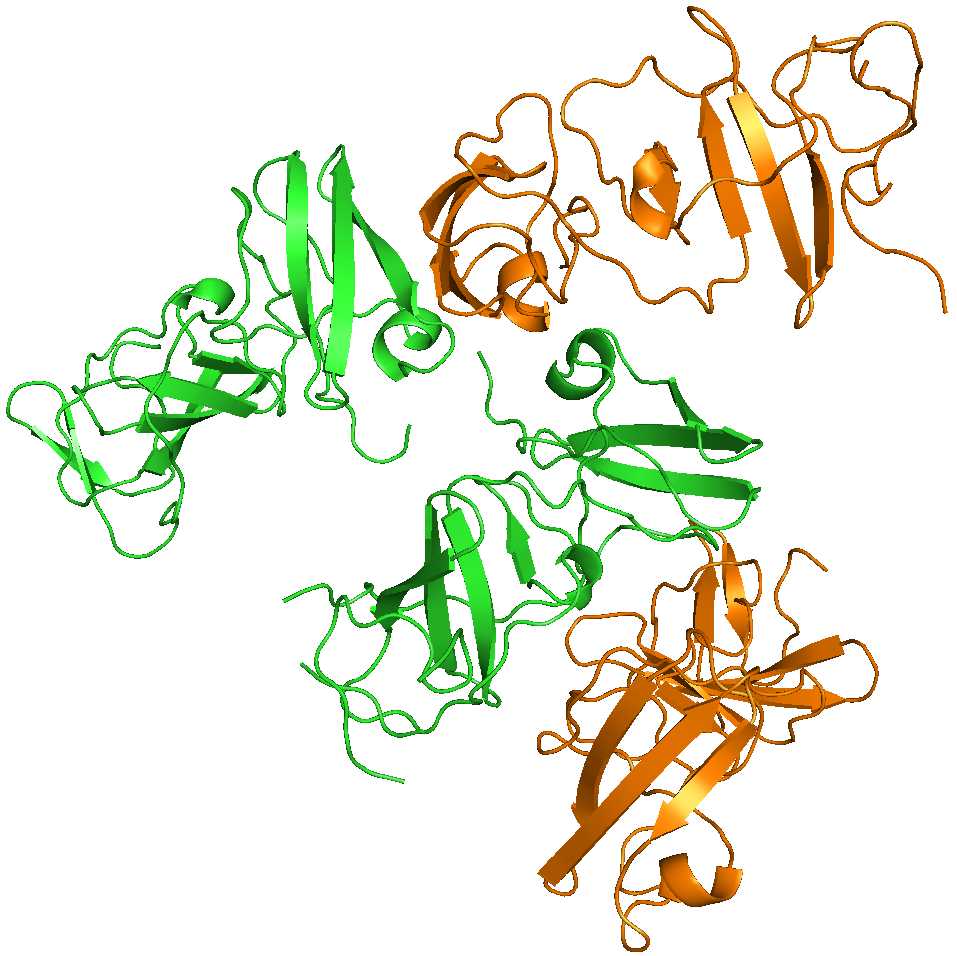  **D**  **C**  **A**  **B** |
| --- |
| **Figure S1.**  The asymmetric unit in the genotype 1a NS5A domain 1 crystal structure. The four monomers A, B, C and D which make up the asymmetric unit comprise two dimeric interfaces, namely a dimer AB of monomers A and B, and dimer CD of monomers C and D. |

| 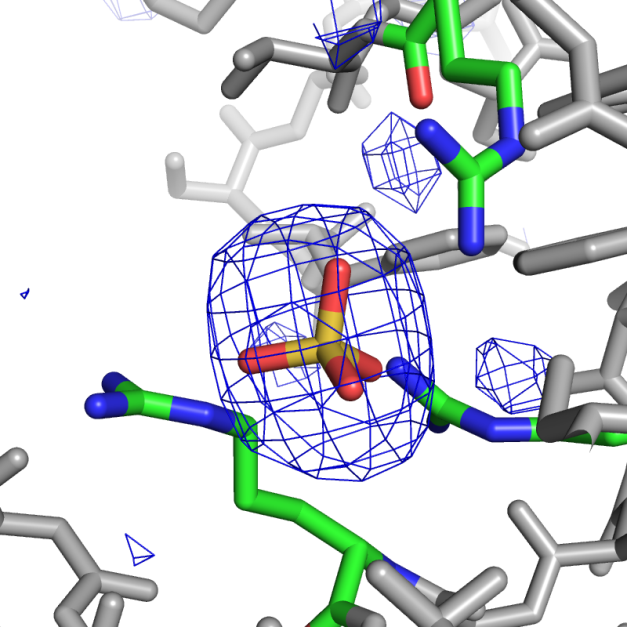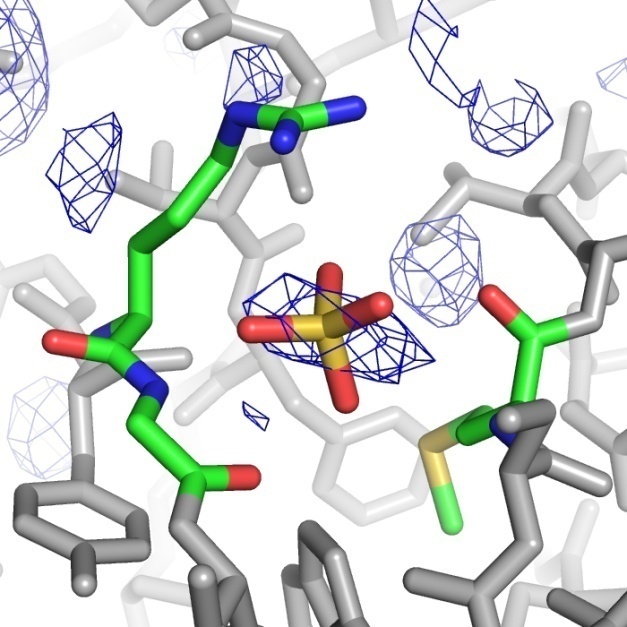  A R81  C R78  A R41  SO_4_^2-^  AM72  AG45  A R44  SO_4_^2-^  **B**  **A** |
| --- |
| **Figure S2.** Sulfate molecules observed in crystals of genotype 1a NS5A domain 1. (**A**) A sulfate ion fits well to electron density difference maps and appears to be coordinated by three arginine residues: Arg 41 and Arg 81 from monomer A, and Arg 78 from chain C.   \|  \| [**http://imolecules3d.wiley.com:8080/imolecules3d/review/hXEcvD1OzRnfVsTtqhModdNGqksbCc3xfVKTI83CDcfZh9A5TJrc5TyYnQo1gtmb728/1411**](http://imolecules3d.wiley.com:8080/imolecules3d/review/hXEcvD1OzRnfVsTtqhModdNGqksbCc3xfVKTI83CDcfZh9A5TJrc5TyYnQo1gtmb728/1411) \| \| --- \| --- \|   (**B**) An additional bound sulfate ion may be present, possibly bound by Arg 44 from chain C and the backbones of Gly 45 and Met 72 from chain A. However, the blob in the electron density difference is smaller than that due to the sulfate ion in A, which leaves the presence of the sulfate ion at this site more ambiguous or perhaps there is a lower occupancy of sulfate ions at this location in the crystal structure. Both electron density difference maps are contoured at a sigma level of 3.0 and carved at 3.0 Å.   \|  \| [**http://imolecules3d.wiley.com:8080/imolecules3d/review/hXEcvD1OzRnfVsTtqhModdNGqksbCc3xfVKTI83CDcfZh9A5TJrc5TyYnQo1gtmb728/1412**](http://imolecules3d.wiley.com:8080/imolecules3d/review/hXEcvD1OzRnfVsTtqhModdNGqksbCc3xfVKTI83CDcfZh9A5TJrc5TyYnQo1gtmb728/1412) \| \| --- \| --- \| |

| 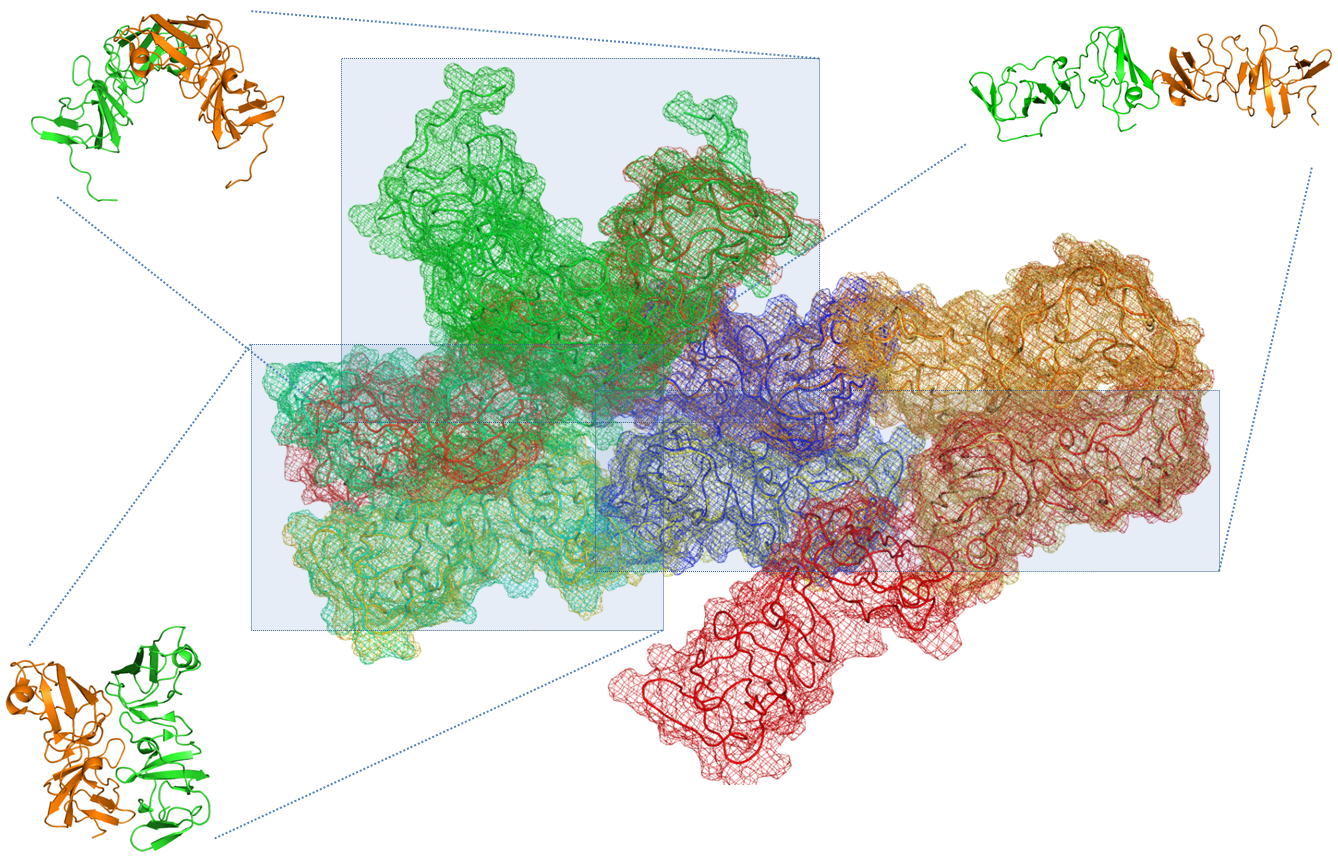  *1b Rice dimer*  *1b Love dimer*  *1a dimer CD* |
| --- |
| **FigureS3.**  Two-dimensional NS5A domain 1 network. Combining more than 2 non-overlapping different monomer-monomer interfaces allows the creation of a theoretical assembly of NS5A domain I proteins. Such a network could potentially form part of the membranous web and explain the multifaceted role of NS5A. |

| Residue Pair | Mean Distance between Residues | |
| --- | --- | --- |
|  | C-monomer | D-monomer |
| Tyr93-Arg56 | 3.52 ± 0.30 | 3.15 ± 0.35 |
| Arg56-Glu62 | 3.66 ± 0.73 | 3.49 ± 0.73 |
| Glu62-DCV | 3.48 ± 0.93 | 3.58 ± 1.05 |
| Tyr93-Glu62 | 7.15 ± 1.16 | 8.04 ± 0.62 |
| Tyr93-DCV | 11.54 ± 1.48 | 12.38 ± 0.85 |

Table S1. : The mean closest heavy atom distances between residues of the communication network that connect Tyr93 with DCV suggest an allosteric mechanism of resistance for this model.
